# Soil acidification and nutrient imbalance mediate fungal community degradation, a key driver of continuous cropping obstacles in *Platycodon grandiflorus*

**DOI:** 10.3389/fmicb.2025.1716243

**Published:** 2025-11-26

**Authors:** Li Li, Hao Yang, Dian Peng, Ruibo Liu

**Affiliations:** College of Biology Pharmacy and Food Engineering, Shangluo University, Shangluo, Shaanxi, China

**Keywords:** *Platycodon grandiflorus*, continuous cropping, rhizosphere fungi, community structure, function

## Abstract

**Introduction:**

Continuous cropping obstacles severely restrict the sustainable cultivation of *Platycodon grandiflorus*. This study aimed to clarify the key drivers underlying these obstacles, focusing on the roles of soil physicochemical properties and rhizosphere fungal community dynamics.

**Methods:**

This study analyzed the growth indicators of *Platycodon grandiflorus*, soil physicochemical properties, and the characteristics and functions of fungal communities under different durations of continuous cropping years.

**Results:**

Continuous cropping significantly inhibited the growth of *Platycodon grandiflorus*. As the continuous cropping years continued, root length, root diameter, yield, and alcohol-soluble extract content decreased, the abundance of Fusarium increased, and root rot was exacerbated. Continuous cropping led to soil acidification, reduced phosphorus and potassium contents, and an imbalance of the fungal community; the abundance of acid-tolerant pathogens increased while beneficial fungi decreased in abundance, with pathotrophic and saprotrophic fungi enriched, resulting in slowed soil carbon turnover and reduced disease resistance.

**Discussion:**

Our findings demonstrate soil acidification and phosphorus/potassium depletion mediate fungal community degradation. Adjusting soil pH, supplementing phosphorus/potassium, and inoculating functional fungi may alleviate obstacles, providing empirical support for sustainable *Platycodon grandiflorus* cultivation

## 1 Introduction

*Platycodon grandiflorus* is a perennial herbaceous medicinal plant in the Campanulaceae family. Its roots have high levels of active ingredients, such as saponins and polysaccharides, which have multiple pharmacological effects, including anti-inflammatory, antioxidant, and immune-regulating properties; accordingly, they are widely used in traditional Chinese medicine and as functional foods (Fu et al., 2025). Owing to the increasing scarcity of wild *Platycodon grandiflorus* resources, the roots are now primarily derived from artificial cultivation. In addition, in recent years, the domestic and foreign demand for this crop has been continuously increasing, while the requirements for its yield have also been increasing. The cultivation of this crop is restricted by the amount of land available, and continuous cropping has become the primary mode of production in various regions. The subsequent continuous cropping obstacles have seriously restricted the sustainable development of this industry (Liu et al., 2025).

The causes of continuous cropping obstacles involve the interaction of multiple factors, such as the deterioration of physicochemical properties of the soil, accumulation of autotoxic chemicals, and the imbalance of microbial communities. Among these problems, the abnormal succession of rhizosphere microbial communities is considered one of the core driving mechanisms (Ding et al., 2024; Lin et al., 2023; Chen et al., 2022). The rhizosphere serves as a key interface for plant–soil–microbe interactions; its microbial communities, particularly fungi, play an irreplaceable role in the transformation of nutrients, inhibition of disease, and regulation of plant secondary metabolism (Pantigoso et al., 2022). Published studies have confirmed that continuous cropping can drive the reconstruction of fungal community structure by changing the rhizosphere microenvironment, e.g., creating an imbalance of nutrients. This imbalance specifically manifests in the enrichment of pathogenic fungi and the reduction of beneficial fungi, thereby disrupting the soil microecological balance and increasing the occurrence of diseases (Song et al., 2022). The application of high-throughput sequencing technology has revealed that continuous cropping often leads to a decrease in fungal diversity and a functional bias toward pathogenic and saprophytic metabolism (Sun et al., 2022). The abundance of pathogenic fungi is significantly positively correlated with the degree of plant disease (Yuan et al., 2020).

Although studies, such as those described above, have provided a basis to understand the microbiological mechanism of continuous cropping obstacles, there is a limited understanding of these for *Platycodon grandiflorus*, a common medicinal plant. The functional transformation pattern of rhizosphere fungal communities during the continuous cropping of this species, the driving environmental factors, and their relationship with medicinal quality remain unclear. Thus, this study utilized continuously cropped *Platycodon grandiflorus* from the Shangluo production area located in the southeastern part of Shaanxi Province, China as the research object and analyzed the changes in the composition, structure, and functional characteristics of the rhizosphere soil fungal communities through high-throughput sequencing. The goals were to clarify the mechanism of imbalance of the fungal community induced by continuous cropping and to provide empirical support to alleviate the obstacles of continuously cropping *Platycodon grandiflorus* and improve the yield and quality of medicinal materials by regulating the microecology of this ecosystem.

## 2 Materials and methods

### 2.1 Overview of the experimental site

This study was conducted in a continuous cropping fixed micro-plot test field in Luo Village, Dazhaoyu Street, Shangzhou District, Shangluo City, Shaanxi Province, China (33°50′44″N, 110°0′14″E), and the geographical location of this study field is depicted in Supplementary Figure 1. The area has an altitude of 752.1 m and a warm temperate semi-humid continental monsoon climate, with annual hours of sunshine of approximately 2,200 h, an average annual temperature of 12.9 °C, and annual precipitation of 720 mm. The soil type is cinnamon soil, which is suitable for the cultivation of *Platycodon grandiflorus*.

### 2.2 Experimental design

In March 2019, a 3-year fallow field that had previously been dedicated to corn (*Zea mays*) was selected as the experimental base. The plots were 36.5 m long and 18.3 m wide, with a total area of 666.7 m^2^. A randomized block design was adopted in this study, and the plots were divided into equal areas of four replicates. Each contained three subplots of 55.6 m^2^ each. The subplots had 1 m gaps between them to reduce the degree of interference. The test materials were 1-year-old seedlings of *Platycodon grandiflorus* with consistent growth that were planted in the same period in March 2019. The plants were spaced at 20 cm × 30 cm intervals. This study established three treatments based on the continuous cropping years of *Platycodon grandiflorus*, as detailed in Supplementary Table 1.

CK (2-year continuous cropping of *Platycodon grandiflorus*): From 2019 to 2022, corn was planted for 4 years; subsequently, *Platycodon grandiflorus* was planted for 2 years (2023–2024).

A (4-year continuous cropping of *Platycodon grandiflorus*): From 2019 to 2020, corn was planted for 2 years; subsequently, *Platycodon grandiflorus* was planted for 4 years (2021–2024).

B (6-year continuous cropping of *Platycodon grandiflorus*): *Platycodon grandiflorus* was planted continuously from 2019 to 2024.

All the treatments were managed similarly. The following amounts of slow-release fertilizer were applied each year: 150 kg/hm^2^ of nitrogen (N), 120 kg/hm^2^ of phosphorous pentoxide (P_2_O_5_), and 90 kg/hm^2^ of potassium oxide (K_2_O). Drip irrigation was used to maintain the content of soil moisture at 20–25%, and the plots were weeded manually to avoid interference between the chemicals.

### 2.3 Collection of samples

The *Platycodon grandiflorus* plants were harvested in October 2024. The five-point sampling method was used to select five plants in each subplot with uniform growth. Samples of the rhizosphere soil were collected by careful excavation of the root system, and the root-shaking method was used to collect the soil that had tightly adhered to the root surface (<2 mm) (Hou et al., 2025). Five samples from each subplot were mixed into one replicate, and each treatment had four replicates. The samples were placed in sterile self-sealing bags, transported back to the laboratory on ice within 24 h, and passed through a 2-mm sieve to remove plant residues and stones (as large debris can cause uneven DNA extraction and reduce sequencing data quality). We used manual gentle shaking (no mechanical vibration) and limited sieving time per sample to ≤ 5 min, thus preventing excessive breakdown of soil aggregates, and then divided into soil samples into two portions. One was stored at –80 °C for high-throughput sequencing, while the other was air-dried naturally in a cool and well-ventilated area to be used for the determination of soil physicochemical properties.

### 2.4 Determination of the growth and quality indicators of *Platycodon grandiflorus*

#### 2.4.1 Growth indicators

The root length (cm) was measured with a ruler. The diameters of the root cap and the middle part of the root were measured with an electronic Vernier caliper, and the average value was taken as the root diameter (mm). The fresh weight was measured on an electronic balance, and the dry weight was determined after the samples were dried to a constant weight in a 60 °C oven. Yield (g/m^2^) was calculated as the dry weight per square meter. The drying rate was computed using Equation 1.


dry⁢weight/fresh⁢weight×100% (1)

#### 2.4.2 Medicinal quality

The quality of medicinal materials in the samples was determined from the content of alcohol-soluble extracts (%) using the hot dipping method described in General Rule 2201 of the *Chinese Pharmacopoeia* (2020 edition) (National Pharmacopoeia Commission, 2020).

### 2.5 Investigation of root rot in *Platycodon grandiflorus*

The occurrence of root rot was determined during the peak period of its incidence in August 2024. The disease grading levels were defined as follows: grade 0 (no disease), grade 1 (< 20% of the root system affected), grade 2 (20–80% of the root system affected), and grade 3 (> 80% of the root system affected). The incidence rate (IR) was calculated using Equation 2.


(number⁢of⁢diseased⁢plants/total⁢number⁢of⁢plants⁢examined)×100% (2)

The disease index (Bi et al., 2023) was determined as per Equation 3.


Σ(numberofdiseasedplantsateachgrade×correspondinggrade)/(3×total⁢number⁢of⁢plants⁢examined)×100% (3)

### 2.6 Determination of the soil physicochemical properties

The following soil physicochemical properties were determined as indicated using previously described methods (Serafim et al., 2023): pH value, potentiometric method [1:2.5 (*w*:*w*), soil–water ratio]; organic matter (OM), potassium dichromate oxidation–external heating method; total nitrogen (TN), Kjeldahl method; total phosphorus (TP), sodium hydroxide fusion–molybdenum antimony anti-colorimetric method; total potassium (TK), sodium hydroxide fusion–flame photometry; available nitrogen (AN): alkali-hydrolyzed diffusion method; available phosphorus, sodium bicarbonate extraction-molybdenum antimony antibody spectrophotometry; sodium bicarbonate extraction, molybdenum antimony anti-colorimetric method; available potassium (AK), ammonium acetate extraction–flame photometry.

### 2.7 Metagenomic sequencing and data analysis

The CTAB method was used to extract the genomic DNA from the soil, and 1% agarose gel electrophoresis was used to detect its integrity and purity. A Covaris M220 focused-ultrasonicator (Covaris, Woburn, MA, United States) was used to fragment the DNA into fragments of 350 bp, which were then sequenced on an Illumina NovaSeq platform (Illumina, San Diego, CA, United States). A PE150 strategy was used to create a paired-end (PE) library.

First, adapter sequences at the 5′ and 3′ ends of the sequencing reads were trimmed using fastp software (Chen et al., 2018) (version 0.20.0).^1^ Subsequently, low-quality reads with a length of < 50 bp after trimming or an average base quality score < 20 were filtered out, retaining only high-quality sequences for subsequent analyses.

The optimized sequences were assembled using MEGAHIT software (Li et al., 2015) (version 1.1.2).^2^ Contigs with a length of ≥ 300 bp were selected from the assembly results as the final assembled products. Open reading frames (ORFs) were predicted from the assembled contigs using Prodigal (Hyatt et al., 2010) (version 2.6.3).^3^ Genes with a nucleic acid length of ≥ 100 bp were filtered and translated into amino acid sequences.

To construct a non-redundant gene set, predicted gene sequences from all samples were clustered using CD-HIT software (Fu et al., 2012) (version 4.7)^4^ with parameters of 90% identity and 90% coverage. The longest gene in each cluster was selected as the representative sequence. High-quality reads from each sample were aligned against the non-redundant gene set using SOAPaligner (Li et al., 2008) (version soap2.21 release)^5^ with a 95% identity threshold, and the abundance of each gene in the corresponding sample was quantified.

For functional annotation and abundance calculation, amino acid sequences of the non-redundant gene set were subjected to BLASTP against the NR database using Diamond (Buchfink et al., 2015) (version 2.0.13)^6^ with an e-value cutoff of 1e^–5^. Taxonomic annotation results were obtained by integrating taxonomic information associated with the Non-redundant (NR) NCBI database, and the abundance of each species was calculated by summing the abundances of genes corresponding to each respective species. Concurrently, Diamond (Buchfink et al., 2015) (version 2.0.13) was used to perform BLASTP of the amino acid sequences against the Kyoto Encyclopedia of Genes and Genomes (KEGG) database (*e*-value = 1e^–5^) to obtain KEGG functional annotations for the genes. The co-occurrence analysis network and functional prediction via FUNGuild were both conducted through data analysis using the online tool of the Majorbio Cloud Platform.^7^

The metagenomic data in this study have been submitted to the NCBI database and are specifically deposited in the NCBI BioProject platform, under accession number PRJNA1335859. The public retrieval link is https://www.ncbi.nlm.nih.gov/bioproject/PRJNA1335859.

### 2.8 Statistical analysis

QIIME v1.9.1 was used to calculate the α-diversity index of the fungal community. The Bray–Curtis distance was used to evaluate the differences in β-diversity with a principal coordinate analysis (PCoA). The data on the measurements are expressed as the mean ± SE values. A two-way analysis of variance (ANOVA) was performed using SPSS 27.0 (IBM Inc., Armonk, NY, United States), and Duncan’s method was used to determine significant differences among treatment means while controlling for multiple comparisons. A threshold of *P* < 0.05 was considered significant. A Pearson correlation analysis (two-tailed test) was used to explore the correlation between indicators. Canoco5 was used for a redundancy analysis (RDA) to analyze the impact of soil physicochemical factors on the fungal community. Origin 2022 (OriginLab, Northampton, MA, United States) was used to render the plots.

## 3 Results and analysis

### 3.1 Summary of metagenomic sequencing

The sequencing data of the soil samples were assessed for their quality using Fastp v. 0.20.0 (Table 1). The total number of valid reads across all the samples reached 5.15 × 10^8^ after quality control. The average valid reads for the first cropping (CK), continuous cropping for one stubble period (A), and continuous cropping for two stubble periods (B) treatment groups were 4.22 × 107, 4.30 × 10^7^, and 4.35 × 10^7^ reads, respectively. An additional analysis showed that all the sequencing read quality scores of the three treatment groups were ≥ 98.5%, and all the base quality scores were ≥ 98.2%. This indicated that the sequencing libraries that were constructed were of high quality and met the requirements for subsequent bioinformatics analyses.

**TABLE 1:** Basic data of metagenomic sequencing under different continuous cropping years of *Platycodon grandiflorus.*

Treatment	Raw reads count (reads)	Clean reads count (reads)	Raw bases (bp)	Clean bases (bp)	Reads retention rate (%)	Bases retention rate (%)
CK	4.28 × 10^7^	4.22 × 10^7^	6.47 × 10^9^	6.36 × 10^9^	98.63	98.30
A	4.36 × 10^7^	4.30 × 10^7^	6.59 × 10^9^	6.47 × 10^9^	98.60	98.28
B	4.42 × 10^7^	4.35 × 10^7^	6.67 × 10^9^	6.55 × 10^9^	98.57	98.21

CK, 2-year continuous cropping of *Platycodon grandiflorus*; A, 4-year continuous cropping of *Platycodon grandiflorus*; B, 6-year continuous cropping of *Platycodon grandiflorus*.

### 3.2 Effects of continuous cropping on the growth and quality indicators of *Platycodon grandiflorus*

**FIGURE 1:**
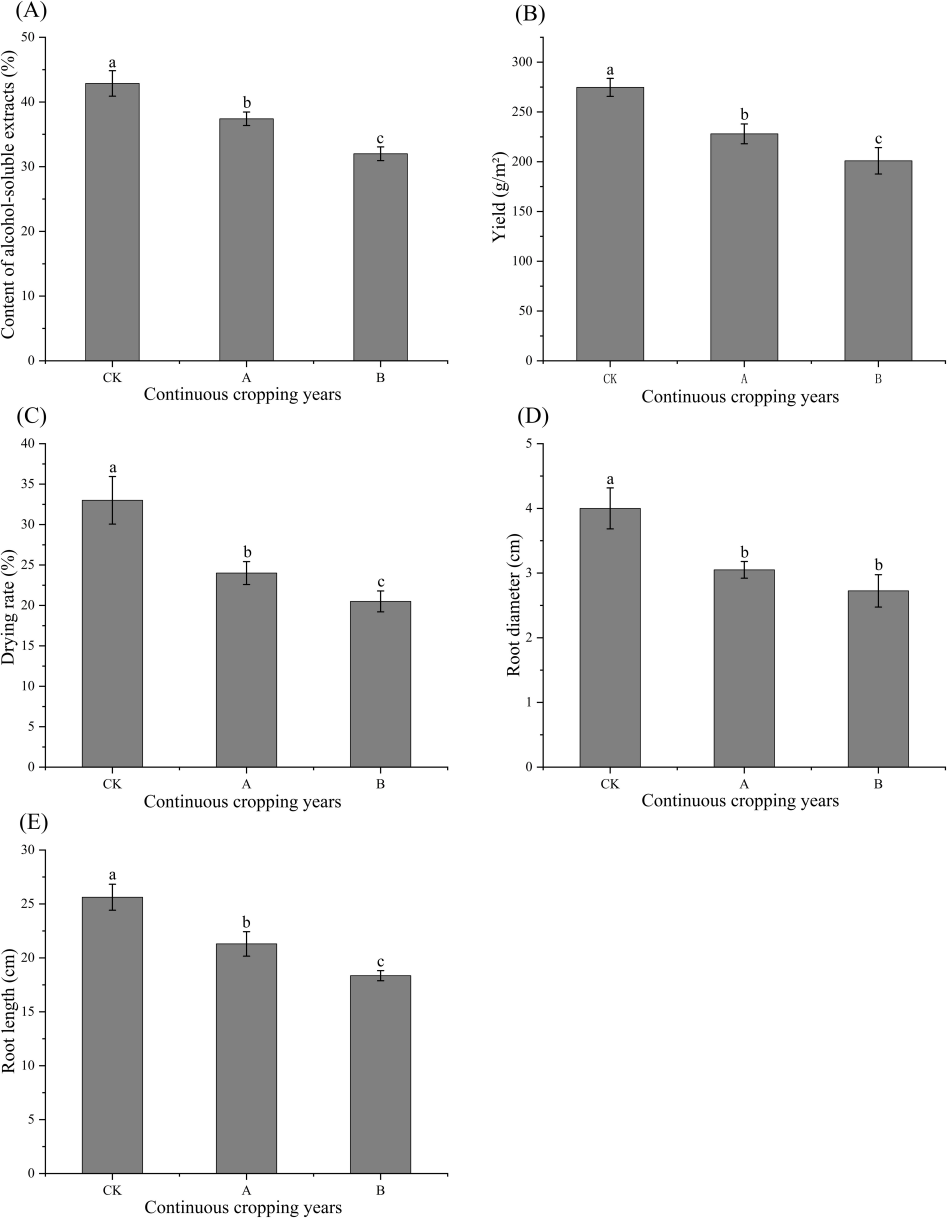
Ethanol extract content **(A)**, yield **(B)**, drying rate **(C)**, root diameter **(D)**, and root length **(E)** of *Platycodon grandiflorus* under different continuous cropping years CK, 2-year continuous cropping of *Platycodon grandiflorus*; A, 4-year continuous cropping of *Platycodon grandiflorus*; B, 6-year continuous cropping of *Platycodon grandiflorus*. Values with different letters indicate significant differences (Duncan’s test *P* = 0.05, *n* = 4).

As shown in Figure 1, continuous cropping significantly inhibited the growth, accumulation of active ingredients, and yields of *Platycodon grandiflorus* (*P* < 0.05). Compared with the first cropping (CK), there were significant reductions in the root diameter and root length when the plants were continuously cropped for one stubble period (A) and continuously cropped for two stubble periods (B). These parameters tended to decrease as the number of continuous cropping years increased. The CK plants had a significantly higher drying rate than that of the A and B treatments. The content of alcohol-soluble extracts, a core indicator of medicinal quality, also significantly decreased with the increase in the years of continuous cropping. The yield was consistent with these indicators, and the yield of the CK was significantly higher than that of the A and B treatments. Thus, all the indicators had their greatest values in the CK treatment followed by the A and then B treatments, which indicated that increased years of continuous cropping more significantly inhibited the growth and quality of the crop.

### 3.3 Effects of continuous cropping on the physicochemical properties of the rhizosphere soil

The analysis of soil physicochemical properties under different continuous cropping years showed that the pH value and content of AK in treatment group B were significantly lower than those in the CK and A groups (Table 2). Continuous cropping led to a significant decrease in the contents of TN and AN, while the content of TK increased significantly. With the increase in continuous cropping years, the contents of TP and AP gradually decreased. The contents of organic matter and TK tended to first increase and then decrease.

**TABLE 2:** Physicochemical properties of rhizosphere soil of *Platycodon grandiflorus* under different continuous cropping years.

Treatment	pH	Organic matter (OM) (g/kg)	Total nitrogen (TN) (g/kg)	Total phosphorus (TP) (g/kg)	Total potassium (TK) (g/kg)	Available nitrogen (AN) (mg/kg)	Available phosphorus (AP) (mg/kg)	Available potassium (AK) (mg/kg)
CK	8.01 ± 0.07a	11.29 ± 0.14b	0.91 ± 0.01a	1.04 ± 0.02a	10.14 ± 0.2c	45.44 ± 0.3a	13.13 ± 0.4a	114.28 ± 1.55a
A	7.95 ± 0.06a	12.76 ± 0.02a	0.78 ± 0.03c	0.74 ± 0.01b	11.79 ± 0.03a	36.53 ± 0.53c	10.54 ± 0.06b	112.08 ± 1.39a
B	7.78 ± 0.03b	10.91 ± 0.26c	0.82 ± 0.02b	0.52 ± 0.01c	11.11 ± 0.02b	37.71 ± 0.4b	7.06 ± 0.09c	90.95 ± 2.19b

The data are the means ± standard error (*n* = 4). Different letters means significantly different based on *P* < 0.05. CK, 2-year continuous cropping of *Platycodon grandiflorus*; a, 4-year continuous cropping of *Platycodon grandiflorus*; b, 6-year continuous cropping of *Platycodon grandiflorus*.

### 3.4 Effects of continuous cropping on the occurrence of root rot and abundance of *Fusarium* in *Platycodon grandiflorus*

**FIGURE 2:**
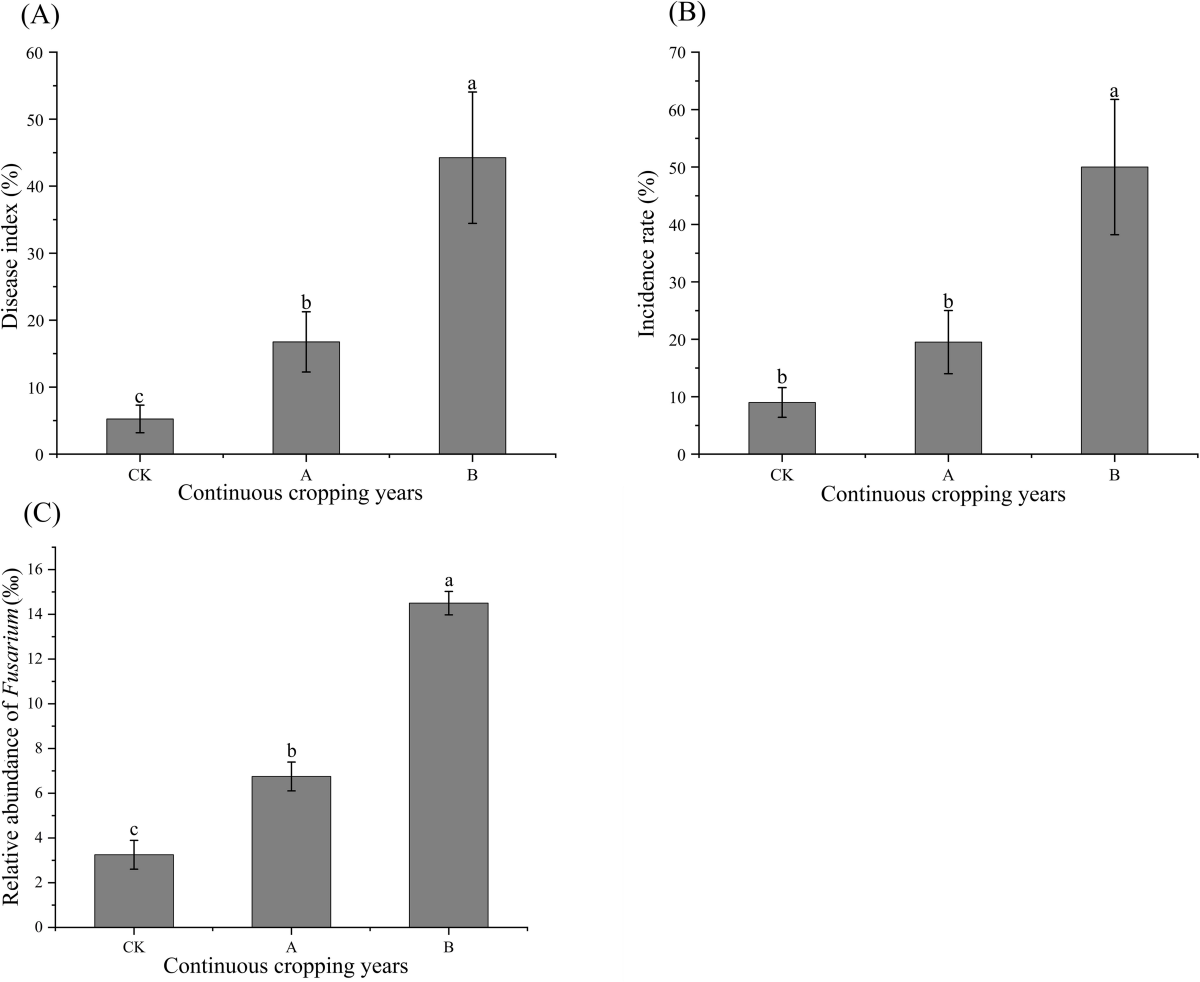
Root rot disease index **(A)**, incidence rate **(B)**, and relative abundance of *Fusarium* **(C)** in *Platycodon grandiflorus* under different continuous cropping years. CK, 2-year continuous cropping of *Platycodon grandiflorus*; A, 4-year continuous cropping of *Platycodon grandiflorus*; B, 6-year continuous cropping of *Platycodon grandiflorus*. Values with different letters indicate significant differences (Duncan’s test *P* = 0.05, *n* = 4).

The analysis of the incidence rate, disease index of root rot, and relative abundance of *Fusarium* in the rhizosphere soil of *Platycodon grandiflorus* under different continuous cropping years showed that continuous cropping significantly increased the occurrence of root rot and led to the enrichment of pathogenic fungi (Figure 2). Both the disease index of root rot and the relative abundance of *Fusarium* increased significantly as the number of years of continuous cropping increased (*P* < 0.05). The incidence rate of continuous cropping for two stubble periods (B) was significantly higher than that of the first cropping (CK) and continuous cropping for one stubble period (A) (*P* < 0.05).

### 3.5 Effects of continuous cropping on the diversity of rhizosphere soil fungal community

#### 3.5.1 α-Diversity of the fungal community

As shown in Table 3, the Coverage index of all the samples was 1.0, which indicated that the sequencing depth had sufficiently covered all the fungal species in the samples. Therefore, the data were highly reliable. A specific diversity index analysis showed that the Chao index of continuous cropping for two stubble periods (B) was significantly higher than that of the first cropping (CK) and continuous cropping for one stubble period (A) (*P* < 0.05). This indicated that the species richness of the fungal community increased significantly as the number of years of continuous cropping increased. The Pielou evenness index and Simpson index did not show significant differences among the three groups (*P* > 0.05), but both indices in group B were the highest; this suggests that the distribution of individual numbers of species in the fungal community was more uniform. These results indicated that continuous cropping primarily changed the α-diversity characteristics of the fungal community by increasing the species richness, while there was relatively little impact on the community evenness.

**TABLE 3:** α-Diversity of fungal communities in rhizosphere soil of *Platycodon grandiflorus* under different continuous cropping years.

Treatment	Chao	Shannon	Simpson	Pielou_e	Coverage
CK	115.25 ± 9.39b	3.65 ± 0.13a	0.06 ± 0.01a	0.77 ± 0.02a	1
A	130.50 ± 21.44b	3.76 ± 0.24b	0.05 ± 0.02a	0.78 ± 0.02a	1
B	142.25 ± 7.41a	3.86 ± 0.08c	0.06 ± 0.01a	0.78 ± 0.01a	1

The data are the means ± standard error (*n* = 4). Different letters means significantly different based on *P* < 0.05. CK, 2-year continuous cropping of *Platycodon grandiflorus*; a, 4-year continuous cropping of *Platycodon grandiflorus*; b, 6-year continuous cropping of *Platycodon grandiflorus*.

#### 3.5.2 β-Diversity of the fungal community

**FIGURE 3:**
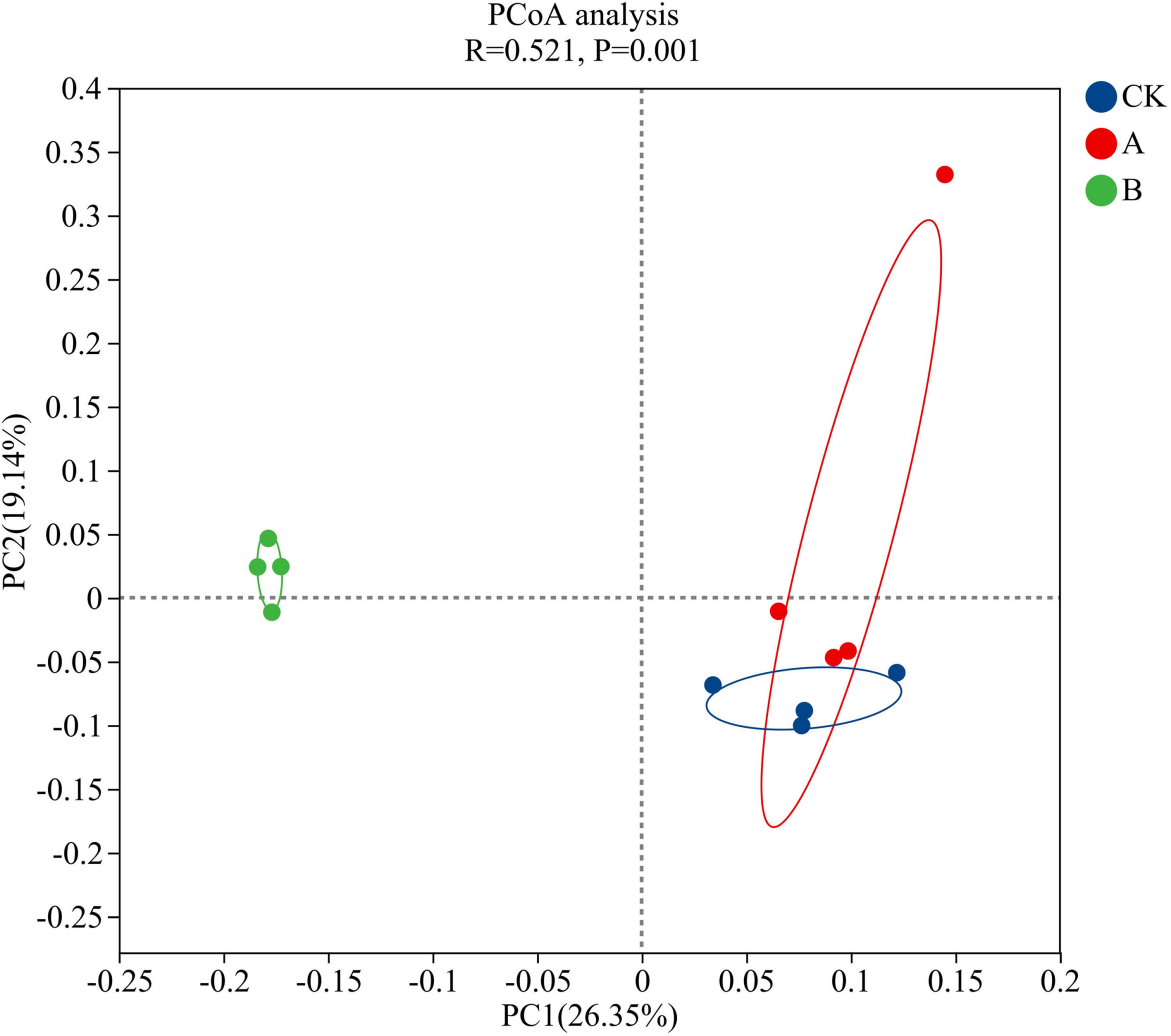
PCoA analysis of fungal community structure in the rhizosphere soil of *Platycodon grandiflorus* under different continuous cropping years. CK, 2-year continuous cropping of *Platycodon grandiflorus*; A, 4-year continuous cropping of *Platycodon grandiflorus*; B, 6-year continuous cropping of *Platycodon grandiflorus*.

The PCoA conducted at the genus level showed that continuous cropping significantly changed the structure of the rhizosphere soil fungal community of *Platycodon grandiflorus* (ANOSIM test, *R* = 0.655, *P* = 0.001) (Figure 3). The first two principal axes together explained 45.49% of the total variation, and the first principal component PC1 contributed 26.35%. The second principal component PC2 only contributed 19.14%. The analysis of the distribution of sample points showed that there was a partial overlap in the coordinate points of the first cropping (CK) and continuous cropping for one stubble period (A). This indicated that their fungal community structures were somewhat similar. However, the sample points of continuous cropping for two stubble periods (B) and the CK showed that there was significant separation on both the PC1 and PC2 axes. This suggested that long-term continuous cropping (B treatment) led to substantial differences in the fungal community structure compared with the first cropping period. Combining this data with the results of the ANOSIM test revealed that the increase in the years of continuous cropping years resulted in a higher degree of differentiation of the fungal community structure.

### 3.6 Effects of continuous cropping on the composition of the rhizosphere soil fungal community

**FIGURE 4:**
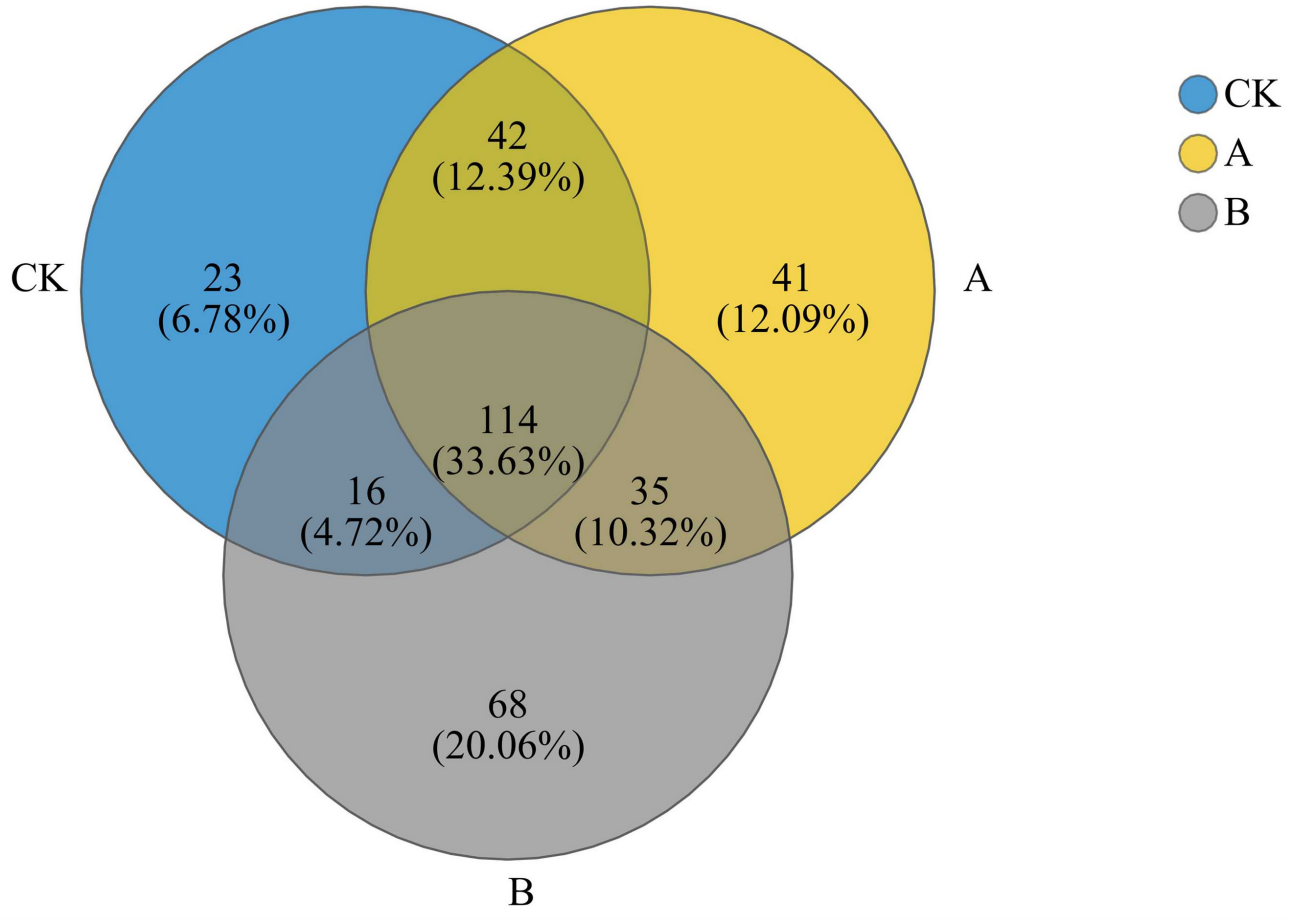
Venn diagram of fungal genera in the rhizosphere soil of *Platycodon grandiflorus* at the genus level under different continuous cropping years. CK, 2-year continuous cropping of *Platycodon grandiflorus*; A, 4-year continuous cropping of *Platycodon grandiflorus*; B, 6-year continuous cropping of *Platycodon grandiflorus*.

As revealed by the Venn diagram in Figure 4, 339 fungal genera were detected in the three treatment groups, and 114 of them were shared by the three groups. This reflects the stability of the core composition of the community. There was a trend in the numbers of shared genera between the groups as the number of years of continuous cropping increased such that groups CK and A shared more genera (156) than did groups A and B (149) or CK and B (130). In addition, the number of unique genera increased significantly with the increase in years of continuous cropping (CK, 23; A, 41; B, 68). These results indicated that continuous cropping significantly changed the composition of rhizosphere fungi at the genus level, and more years of continuous cropping resulted in a higher degree of differentiation of the unique groups in the community.

**FIGURE 5:**
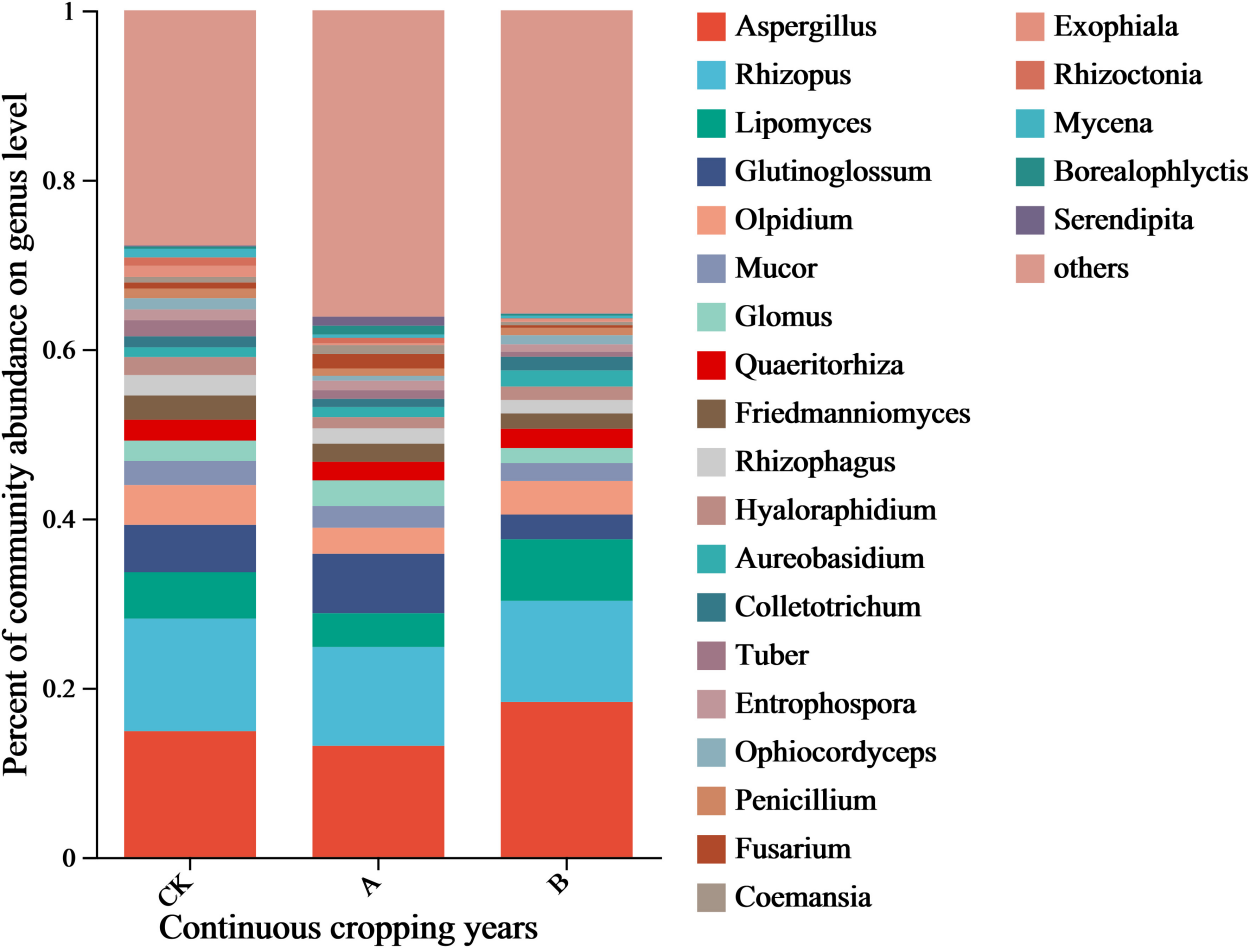
Relative abundance of fungal genera in the rhizosphere soil of *Platycodon grandiflorus* at the genus level under different continuous cropping years. CK, 2-year continuous cropping of *Platycodon grandiflorus*; A, 4-year continuous cropping of *Platycodon grandiflorus*; B, 6-year continuous cropping of *Platycodon grandiflorus*.

The species annotation results of the NR database enabled an analysis of the dominant genera in the community (relative abundance > 1%). This data revealed that *Aspergillus* and *Rhizopus* were the core dominant groups (Figure 5), but their responses to continuous cropping differed. The relative abundance of *Aspergillus* increased with the years of continuous cropping (CK, 14.86%; A, 13.13%; B, 18.81%) and was the highest in treatment group B. *Rhizopus* showed the opposite trend. It was the most abundant in the CK group (13.29%) and decreased to 11.66% and 11.94% in the A and B groups, respectively. There were 20 other dominant genera, including *Lipomyces, Glutinoglossum, Olpidium, Mucor*, and *Glomus*, with relative abundances of 1–5%. Among them, there was a positive correlation in the changes in abundance of genera of pathogens, such as *Fusarium* and *Colletotrichum*, which were positively correlated with the continuous cropping years. In contrast, there was a decrease in the abundance of the genera of symbiotic fungi, including *Glomus* and *Glutinoglossum*.

**FIGURE 6:**
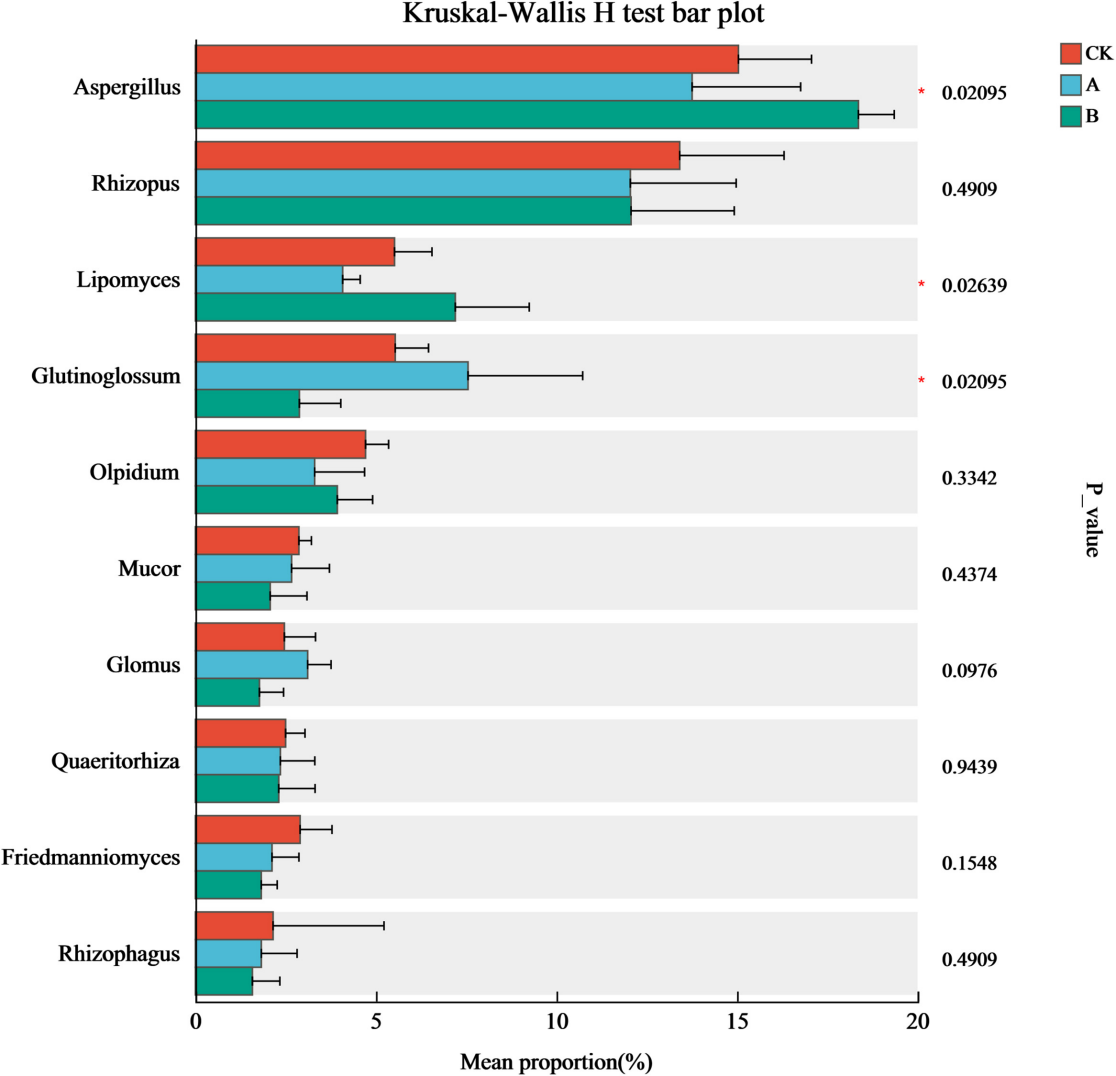
Analysis of intergroup differences in species in the rhizosphere soil of *Platycodon grandiflorus* at the genus level under different continuous cropping years. CK, 2-year continuous cropping of *Platycodon grandiflorus*; A, 4-year continuous cropping of *Platycodon grandiflorus*; B, 6-year continuous cropping of *Platycodon grandiflorus*.

The impact of continuous cropping on the core dominant fungi was clarified by conducting tests of the inter-group differences among the top 10 genera in terms of relative abundance in the community (Figure 6). There were significant differences in *Aspergillus, Glutinoglossum*, and *Lipomyces* abundance among the different treatments (*P* < 0.05). In particular, there was a clear trend in the changes in the relative abundances of *Aspergillus* and *Lipomyces* among the different treatments. They were more abundant in the treatment with continuous cropping for two stubble periods (B) than in the first cropping period (CK) and continuous cropping for one stubble period (A). This trend continued with the extension of years of continuous cropping. *Glutinoglossum* had a higher relative abundance in group A. This decreased significantly in group B and showed a characteristic pattern of initially increasing and then decreasing. This genus was significantly less abundant in group B than in group CK. These results indicated that these three types of fungi were the most sensitive to continuous cropping and might be the key groups that drive the reconstruction of the rhizosphere fungal community structure.

### 3.7 Effects of continuous cropping on the function of the rhizosphere soil fungal community

**FIGURE 7:**
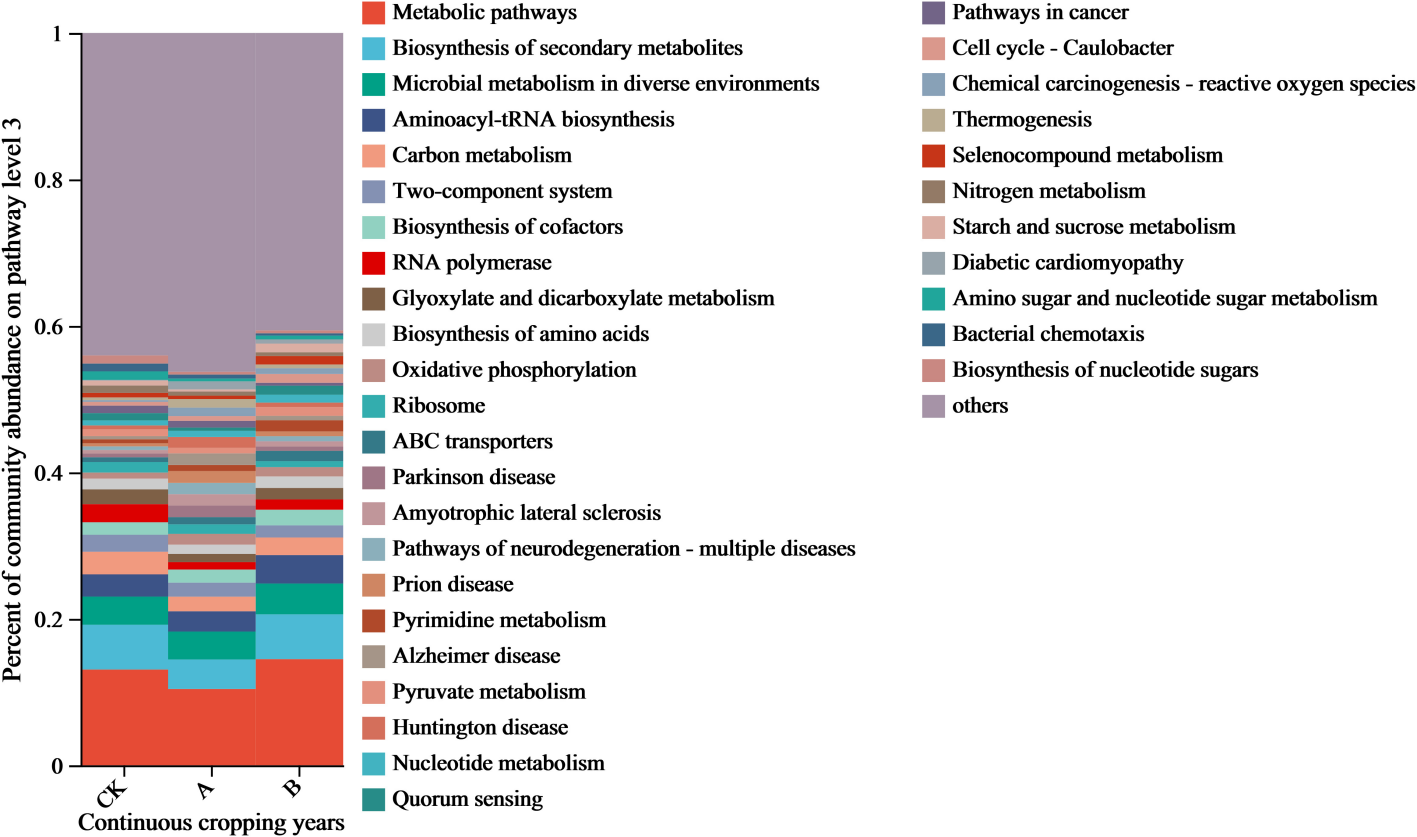
Functional gene composition of fungal communities in the rhizosphere soil of *Platycodon grandiflorus* under different continuous cropping years. CK, 2-year continuous cropping of *Platycodon grandiflorus*; A, 4-year continuous cropping of *Platycodon grandiflorus*; B, 6-year continuous cropping of *Platycodon grandiflorus*.

The regulatory effect of continuous cropping on the functional characteristics of the fungal community was examined by an annotation analysis of the functional gene sequences based on the KEGG database. As shown in Figure 7, there was a consistent dominant trend in the distribution of relative abundances of the functional genes in the different treatment groups. Metabolism pathway genes accounted for the highest proportion (10.42–14.50%). This indicated that the metabolic activities of rhizosphere fungi were always in the core position. This was followed by the Biosynthesis of secondary metabolites, Microbial metabolism in diverse environments, and Aminoacyl-tRNA biosynthesis, which accounted for 9.68–16.26% of the total proportion of these three types of genes. The remaining primary functional genes were Carbon metabolism and Biosynthesis of cofactors among others, in their order of abundance. These genes accounted for 1–5% of the total number of genes.

**FIGURE 8:**
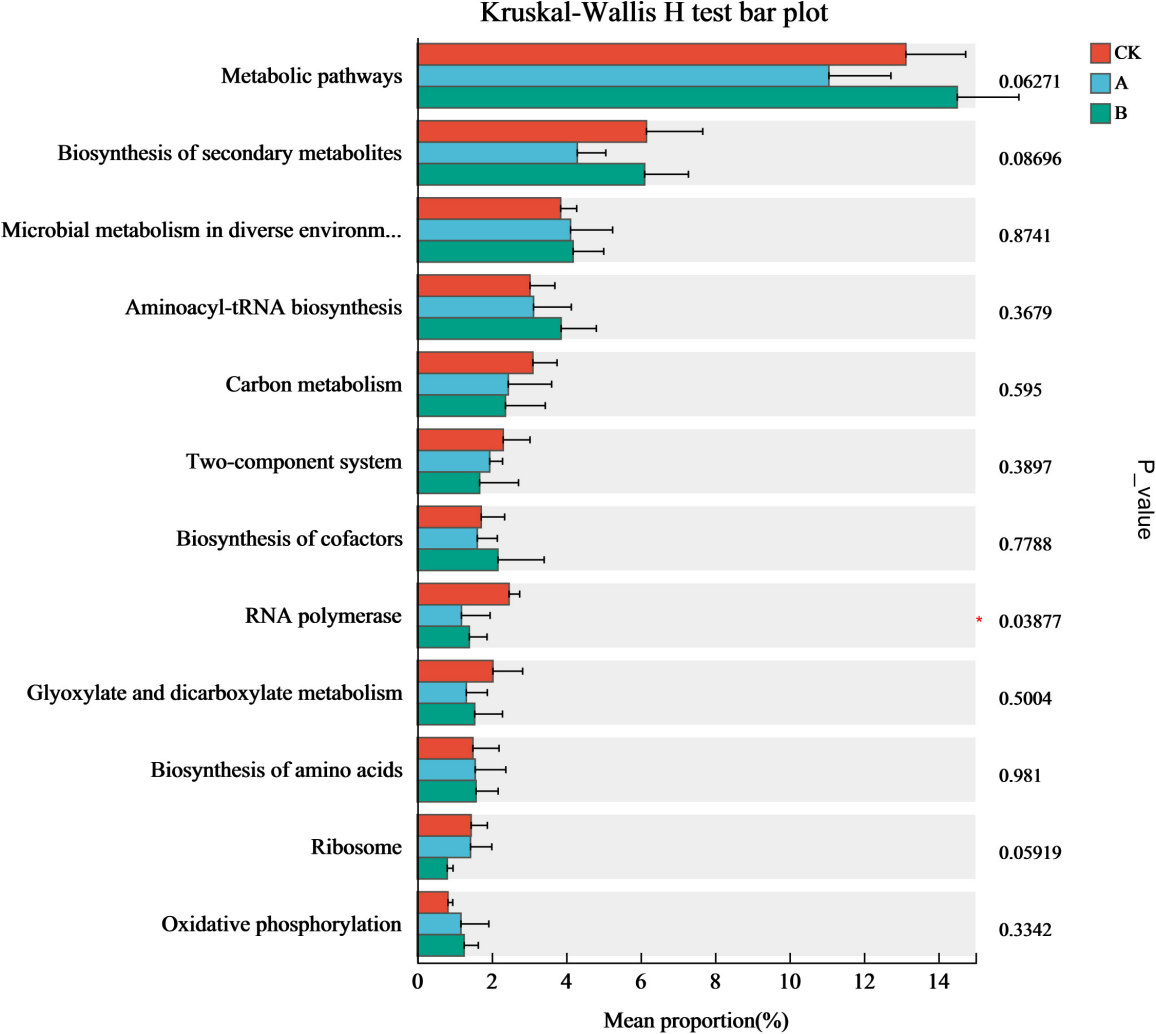
Differential expression of functional genes in fungal communities from the rhizosphere soil of *Platycodon grandiflorus* under different continuous cropping years. CK, 2-year continuous cropping of *Platycodon grandiflorus*; A, 4-year continuous cropping of *Platycodon grandiflorus*; B, 6-year continuous cropping of *Platycodon grandiflorus*.

The analysis of the inter-group difference tests based on the relative abundance of the top 12 functional genes showed that only the relative abundance of RNA Polymerase genes changed significantly in the continuous cropping treatments (Figure 8). The abundances of RNA polymerase genes in continuous cropping for one stubble period (A) and continuous cropping for two stubble periods (B) were 51.84 and 43.26% lower than those in the first cropping (CK), respectively (*P* < 0.05), and tended to decrease as the number of years of continuous cropping increased. This suggested that continuous cropping might drive the fungal community to adjust its metabolic strategy through adaptive evolution by inhibiting the functions of the fungal community that are related to transcription, thereby changing the ecological function of the rhizosphere soil.

Based on FUNGuild functional prediction, the analysis results of fungal trophic modes in soils under 2-year (CK), 4-year (A), and 6-year (B) continuous cropping showed (Supplementary Table 2) that there were significant differences in the composition of fungal functional groups among different continuous cropping years. The abundance of pathotrophs was the highest in the 6-year continuous cropping (B) soil, which was significantly higher than that in the 2-year and 4-year continuous cropping soils (*P* < 0.05). The abundance of Saprotrophs in the 4-year (A) and 6-year continuous cropping soils was significantly higher than that in the 2-year continuous cropping (CK) soil (*P* < 0.05). There was no significant difference in the abundance of symbiotrophs among the three groups.

### 3.8 Effects of continuous cropping on the marker functions of the rhizosphere soil fungi

**FIGURE 9:**
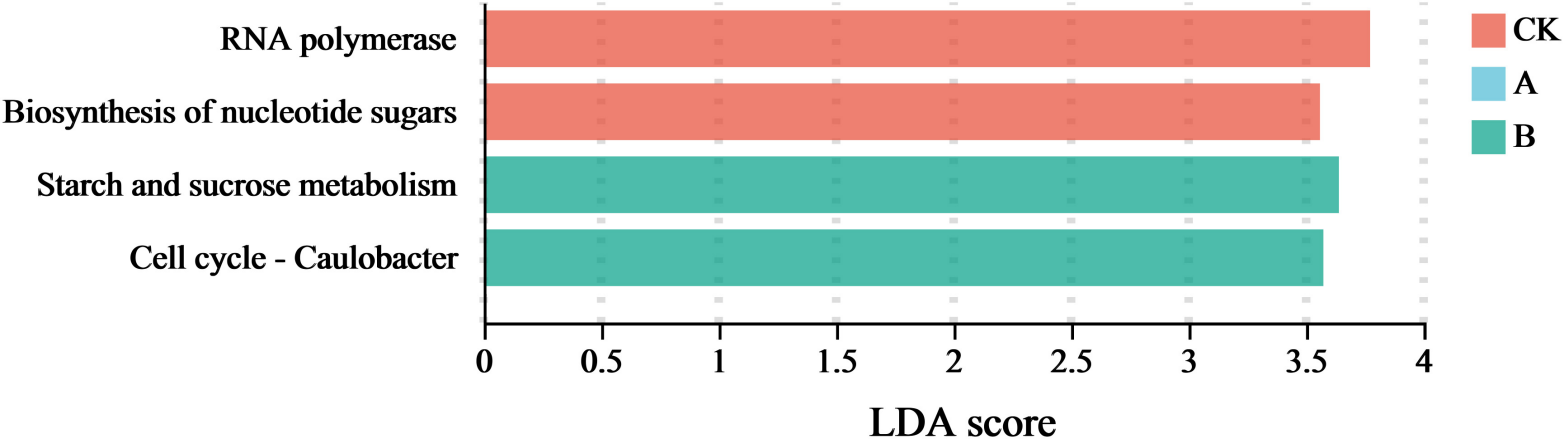
Signature functions of fungal communities in the rhizosphere soil of *Platycodon grandiflorus* under different continuous cropping years. CK, 2-year continuous cropping of *Platycodon grandiflorus*; A, 4-year continuous cropping of *Platycodon grandiflorus*; B, 6-year continuous cropping of *Platycodon grandiflorus*.

The specific differentiation of fungal community functions driven by continuous cropping was analyzed in more detail by combining a linear discriminant analysis (LDA) with a linear discriminant analysis effect size analysis (LEfSe) (*P* < 0.05). This approach was utilized to screen the functional characteristics with significant marker significance in the different treatment groups. An LDA score ≥ 3.5 was defined as biologically significant. As shown in Figure 9, a higher LDA score on the horizontal axis indicated the presence of a more significant difference in the relative abundance of the function in the corresponding group. Thus, these values indicated a greater contribution to functional differentiation between the groups.

This study showed that a total of five marker functions with significant differences were detected in the treatment groups. Among them, the first cropping (CK) group was enriched with two marker functions, namely RNA polymerase and Biosynthesis of nucleotide sugar. Both of these functions were related to basic metabolic regulation. This genus had the highest LDA score in this analysis. This suggests that short-term continuous cropping might induce the abnormal expression of functions related to microbial interactions. The continuous cropping for two stubble periods (B) was enriched with two marker functions, among which Starch and sucrose metabolism and Cell cycle - *Caulobacter* related functions had the highest LDA scores. This indicated that long-term continuous cropping significantly enhanced the carbohydrate degradation and cell proliferation-related functions of the fungal community. These results revealed that continuous cropping gradually reshaped the marker functions of the fungal community by shifting them from basic metabolism dominance (CK) to abnormal microbial interactions (A) and enhanced carbon metabolism (B), which was highly consistent with the trend of changes in the community composition and functional genes described earlier.

### 3.9 Redundancy analysis of the species composition of the fungal community and the soil physicochemical properties

**FIGURE 10:**
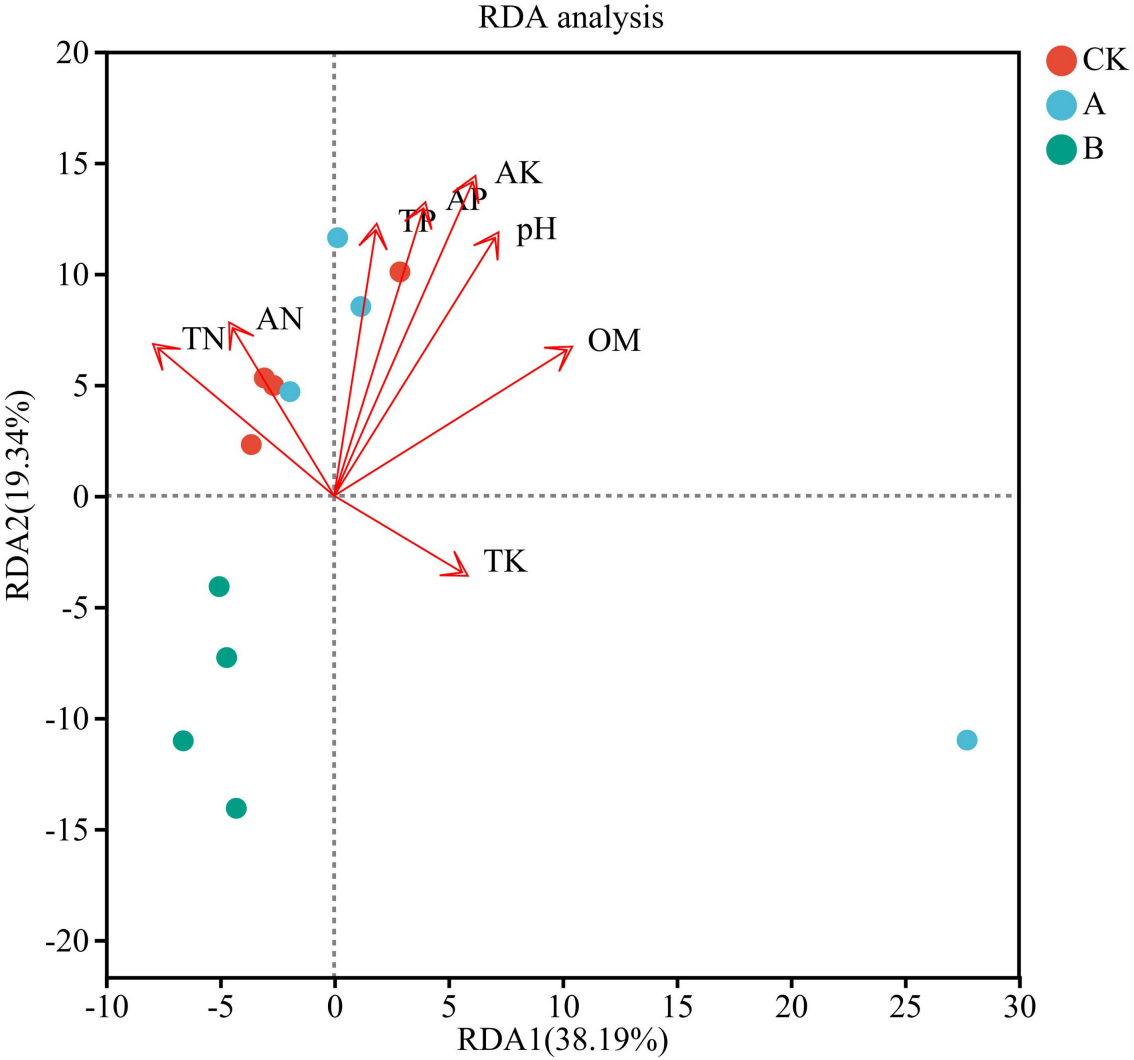
Redundancy analysis (RDA) showing the correlation between soil physicochemical properties and fungal communities in the rhizosphere soil of *Platycodon grandiflorus* under different continuous cropping years. CK, 2-year continuous cropping of *Platycodon grandiflorus*; A, 4-year continuous cropping of *Platycodon grandiflorus*; B, 6-year continuous cropping of *Platycodon grandiflorus*.

The RDA resulted indicated that the soil physicochemical factors collectively explained 57.53% of the variation in fungal community species (Figure 10). The first axis (RDA1) contributed 38.19%, and the second axis (RDA2) contributed 19.34%, which indicated that the soil environmental factors were important drivers of changes in the composition of the rhizosphere fungal community. Specifically, sample points shifted progressively toward the positive end of RDA1 with increasing cropping years, consistent with the directional vectors of key edaphic factors—namely available potassium (AK), pH, and available phosphorus (AP)—in the RDA biplot.

In terms of factor contributions, AK (*r*^2^ = 0.8404, *P* < 0.001), pH (*r*^2^ = 0.6656, *P* = 0.007), and AP (*r*^2^ = 0.6350, *P* = 0.008) emerged as primary drivers of the variance associated with cropping duration. Extended continuous cropping led to significant reductions in soil AK and AP contents, coupled with a reduction in pH values toward acidity; these changes directly induced parallel shifts in the target variables along RDA1. However, weakly influential factors such as total nitrogen (TN, *r*^2^ = 0.2864, *P* = 0.197) and total potassium (TK, *r*^2^ = 0.1202, *P* = 0.640) showed minimal variation across cropping durations, with their sample distributions along RDA2 lacking a clear temporal pattern—further confirming their limited role in mediating the observed variance in target variables.

Notably, samples from treatment B clustered near the extreme positive end of RDA1, exhibiting the strongest associations with AK and pH, and the target variables in this region of the RDA plot were most severely constrained by these factors. Conversely, samples from treatment CK were distributed toward the negative end of RDA1 values, exhibiting greater sensitivity to weaker factors like TN and ammonium nitrogen (AN), and the target variables under this regime showed the most favorable performance.

### 3.10 Pearson correlation analysis

**FIGURE 11:**
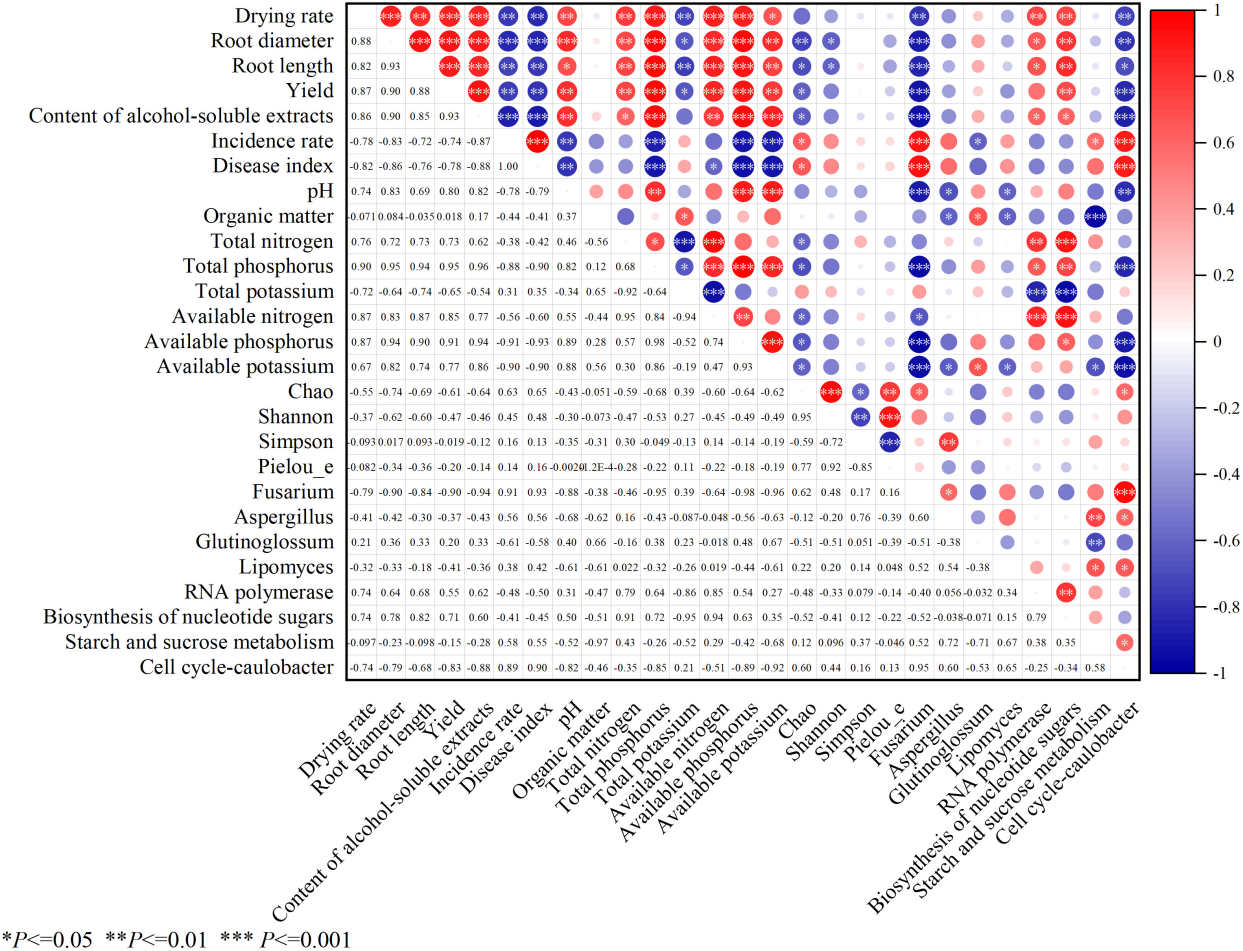
Pearson correlation analysis heatmap: associations among the biomass indicators (root diameter, root length, drying rate, yield, and alcohol-soluble extracts) of *Platycodon grandiflorus*, soil physicochemical properties (pH, total nitrogen, total phosphorus, total potassium, available nitrogen, available phosphorus, available potassium, and organic matter), root rot parameters (incidence rate, and disease index), fungal community diversity indices (Chao, Shannon, Pielou_e and Simpson indices), abundance of pathogenic fungi (*Fusarium*), abundance of intergroup differences in species and marker functional gene abundances.

Pearson correlation analysis was used to systematically explore the associations between the biomass indicators of *Platycodon grandiflorus*, soil physicochemical properties (pH, TN, TP, AN, AP, AK, TK, and OM), root rot parameters (incidence rate, IR, and disease index, DI), fungal community diversity indices (Chao, Shannon, and Simpson indices), abundance of pathogenic fungi (*Fusarium*), and marker functional gene abundances (Figure 11).

#### 3.10.1 Associations between biomass, environmental factors, and diseases

The indicators of *Platycodon grandiflorus* related to the biomass, including drying rate, root length, root diameter, yield, and alcohol-soluble extracts, were significantly positively correlated with the soil pH, TN, TP, AN, AP, and AK (*P* < 0.05) but significantly negatively correlated with TK (*P* < 0.05) (Table 4). These indicators were extremely significantly negatively correlated with the IR and DI of root rot and the relative abundance of *Fusarium* (*P* < 0.01). This indicates that the increase in the contents of N, P, and K in the soil, but not TK, can promote the growth of and improve the quality of *Platycodon grandiflorus*, while the enhancement in the incidence of diseases and the enrichment of pathogenic fungi significantly inhibit its growth.

**TABLE 4:** Data of RDA analysis on the species composition of the fungal community and the soil physicochemical properties.

Environmental factors	RDA1 (%)	RDA2 (%)	*r* ^2^	*P*
pH	55.76	83.00	0.6656	0.007
OM	80.71	59.04	0.5254	0.019
TN	–76.42	64.50	0.2864	0.197
TP	26.65	96.38	0.4924	0.031
TK	88.03	–47.44	0.1202	0.640
AN	–43.26	90.16	0.2174	0.327
AP	38.11	92.45	0.6350	0.008
AK	46.35	88.60	0.8404	0.001

OM, organic matter; TN, total nitrogen; TP, total phosphorus; TK, total potassium; AN, available nitrogen; AP, available phosphorus; AK, available potassium.

#### 3.10.2 Correlations with diversity indices

The Chao index (reflecting species richness) was significantly negatively correlated with the TN, TP, AN, AP, and AK values of the soil and the drying rate, root length, yield, and alcohol-soluble extracts of *Platycodon grandiflorus* (*P* < 0.05) but significantly positively correlated with IR, DI, *Fusarium* abundance, and the abundance of “Cell cycle - *Caulobacter*” functional genes (*P* < 0.05). These findings suggest that high species richness may be related to a reduction in the content of soil nutrients and increase in diseases. The Shannon index was significantly negatively correlated with the DR and RL values (*P* < 0.05).

#### 3.10.3 Association patterns of the dominant fungal genera

The relative abundance of *Aspergillus* was positively correlated with the Simpson index and *Fusarium* abundance, significantly negatively correlated with the soil pH, OM, and AK values (*P* < 0.05), and positively correlated with the abundances of “Starch and sucrose metabolism” and “Cell cycle - *Caulobacter*” functional genes. This indicates that the enrichment of *Aspergillus* may be related to the decline in soil fertility and the enhancement of carbon metabolism functions. The relative abundance of *Glutinoglossum* was significantly positively correlated with the OM and AK values (*P* < 0.05) and significantly negatively correlated with the IR value (*P* < 0.05), suggesting that this genus may have a potential role in inhibiting diseases. The relative abundance of *Lipomyces* was significantly negatively correlated with the pH, OM, and AK values (*P* < 0.05) and significantly positively correlated with the abundances of “Starch and sucrose metabolism” and “Cell cycle - *Caulobacter*” functional genes (*P* < 0.05). Its ecological role is similar to that of *Aspergillus*; both tend to be enriched in continuously cropped soil that had degraded.

#### 3.10.4 Association network of the marker functional genes

The abundances of “RNA polymerase” and “Biosynthesis of nucleotide sugars” functional genes was significantly positively correlated with the *Platycodon grandiflorus* biomass indicators (RT, RL and DR among others) and the TN and TP values of the soil (*P* < 0.05), indicating that the enhancement of basic metabolic functions can promote the growth of *Platycodon grandiflorus*.

The abundance of “Starch and sucrose metabolism” functional genes was significantly negatively correlated with the OM and AK values (*P* < 0.05) and significantly positively correlated with the IR value and abundance of “Cell cycle—*Caulobacter*” functional genes (*P* < 0.05). The abundance of “Cell cycle—*Caulobacter*” functional genes was significantly negatively correlated with the soil pH, TP, AP, AK, and the biomass of *Platycodon grandiflorus* (*P* < 0.05) and highly significantly positively correlated with the IR and DI values (*P* < 0.01). These findings indicated that abnormal carbon metabolism functions and the enrichment of genes related to the proliferation of cells may be important functional markers of the increase in diseases that was associated with continuous cropping.

### 3.11 Co-occurrence network analysis

In this study, a co-occurrence network was constructed between different treatment groups (CK in green, A in red, B in blue) and fungal genera (purple nodes labeled “Genus”) (Supplementary Figure 2). Many significant association edges were detected in the overall network. In terms of node association strength, all three treatment groups (CK, A, and B) were associated with numerous fungal genera. Among these associations, treatment group A (red node) had the most associations with fungal genera, corresponding to the largest number of fungal genera (50). Treatment groups CK (green node) and B (blue node) also had strong associations with multiple fungal genera. In terms of specific taxa, *Aspergillus* was a key group in the network, with direct associations with all treatment groups, while *Atractiella* was only associated with treatment group A. Additionally, *Pisolithus* was associated with treatment groups A and B, and *Bacidia* was associated with treatment groups CK and A, indicating the selective response of different fungal genera to the three treatments.

## 4 Discussion

This study utilized a multi-dimensional analysis to reveal the mechanism of degradation of the rhizosphere microecosystem of *Platycodon grandiflorus* under the continuous cropping. The core of this mechanism is a chain reaction of soil nutrient pool imbalances leading to potential functional shifts in the fungal community, thereby causing a synergistic decrease in plant growth and defense, which involves complex interactions among soil physicochemical properties, microbial interactions, and responses of plant physiology.

### 4.1 Inhibitory effects of continuous cropping on the growth and quality of *Platycodon grandiflorus* and their driving factors

Continuous cropping significantly reduced the root length, root diameter, drying rate, and yield of *Platycodon grandiflorus*. Additionally, the content of alcohol-soluble extracts, which is a core indicator of the medicinal quality of this crop, showed a gradual decrease with the number of continuous cropping years. This is consistent with the common growth inhibition–quality degradation phenomenon observed in the continuous cropping of medicinal plants, such as the observed decrease in the content of ferulic acid caused by the continuous cropping of dong quai (*Angelica sinensis*) (Ahsan et al., 2024) and the obstruction in the biosynthesis of saponin caused by the continuous cropping of Chinese ginseng (*Panax notoginseng*) (Chen et al., 2024). A correlation analysis showed that these indicators were significantly positively correlated with the contents of TN, TP, AN, AP, and AK in the soil and significantly negatively correlated with the fungal Chao index and the abundance of *Fusarium*. These findings suggest that the targeted nutrient deficiency and enrichment of pathogenic fungi are dual driving factors.

It is notable that the extremely significant positive correlation between the abundance of *Fusarium* and the IR and disease index of root rot (*P* < 0.01) confirms its pathogenicity as the causal agent of root rot in *Platycodon grandiflorus*; some strains of this genus can secrete toxins, such as fusaric acid, that damage the integrity of root cell membranes and inhibit the absorption of nutrients by plants (Pan et al., 2023). Simultaneously, the increase in fungal richness, such as the increase in Chao index, caused by continuous cropping is not a manifestation of community stability but the result of the occupation of ecological niches by opportunistic pathogens. This is consistent with the view that the increase in microbial diversity associated with continuous cropping obstacles is often accompanied by functional redundancy (Li et al., 2024).

In the present study, the relative abundance of *Fusarium* showed a significantly negative correlation with soil pH, total phosphorus (TP), available nitrogen (AN), available phosphorus (AP), and available potassium (AK), while a significantly positive correlation was observed with the relative abundance of *Aspergillus* and cell cycle-*Caulobacter* functional genes (Figure 11). As a heterotrophic fungus, *Fusarium* can prioritize the acquisition of available nutrients via its hyphal network (Yan et al., 2023). This competitive advantage not only leads to nutrient competition with crops but also impinges upon the ecological niches of beneficial microorganisms such as nitrogen-fixing bacteria and phosphate-solubilizing bacteria, thereby impairing the soil’s nutrient supply capacity (Armer et al., 2024; Wang et al., 2022; Yan et al., 2022). Meanwhile, long-term soil acidification results in nutrient leaching or immobilization, further reducing nutrient availability (Zama et al., 2022). Previous research has demonstrated that under nutrient competition pressure, *Fusarium* can secrete toxins that inhibit the activities of surrounding microorganisms and crop roots (Bugingo et al., 2025). This not only helps *Fusarium* consolidate its ecological niche advantage but also indirectly disrupts the balance of soil biological interactions. Both *Fusarium* and *Aspergillus* are efficient decomposing fungi. The synchronous increase in their abundances accelerates the decomposition of soil organic matter; while this process releases nutrients in the short term, it can cause the long-term depletion of soil carbon pools (Sharma et al., 2024). Consequently, the stability of soil aggregates and the water- and fertilizer-retention capacities of soil are reduced. Additionally, organic acid production during decomposition exacerbates soil acidification, further driving soil degradation (Li et al., 2023; Meng et al., 2023).

### 4.2 Degradation of the soil physicochemical properties and their regulatory effects on the fungal community

The RDA results indicated that pH, TP, AP, and AK are the key environmental factors that drive the differentiation of the fungal community, and their dynamic changes thus constitute the soil signals of challenges posted by continuous cropping. Continuous cropping led to a significant decrease in the soil pH (acidification) and a continuous decline in the contents of TP, AP, and AK. This process is closely related to the preference of *Platycodon grandiflorus* for absorbing nutrients and the effect of root exudates. The high demand of this plant for P and K can lead to the depletion of the soil pool after long-term absorption. In addition, the organic acids that they secrete, such as citric acid, can activate a portion of the mineral P but increase the acidification of soil. Therefore, this forms a vicious cycle of a decrease in the availability of P and K and intensified acidification (Delalandre et al., 2025; Zhang et al., 2022).

Soil acidification has a selective impact on the fungal community. The abundance of acid-tolerant pathogens, such as *Aspergillus* and *Lipomyces*, increases, while there is a decrease in beneficial fungi that prefer neutral to alkaline environments, such as *Glutinoglossum*. This selective enrichment may be achieved through two pathways. One is acidic conditions promoting the germination of pathogenic spores and the growth of hyphae (Ni et al., 2023). The other is acidification, inhibiting the activity of soil enzymes, such as urease, thus reducing the efficiency of the conversion of N and P and indirectly favoring fungi that are tolerant to low levels of nutrients (Shi et al., 2023). In addition, the positive correlation between the content of AK and the abundance of *Glutinoglossum* suggests that this genus may be involved in the activation of mineralized K by secreting enzymes that solubilize K, such as β-glucosidase; thus, the reduction in the abundance of this genus further exacerbates the shortage of K (Rajawat et al., 2019).

### 4.3 Metabolic mechanism of the functional transformation of fungal community

The KEGG and LEfSe functional analyses revealed that continuous cropping led to a shift in fungal functions from “basic metabolism dominance” to “disease association and abnormal carbon metabolism,” which was highly synchronized with changes in community composition. As the core enzyme involved in transcription, the expression of RNA polymerase genes is regulated by TN and TP. The decrease in abundance directly reflects the decrease in the metabolic activity of microorganisms, which is consistent with the depletion of N and P in the soil. The enrichment of starch and sucrose metabolism genes may be related to the increased demand of pathogenic fungi for decomposing plant residues. The amylase such fungi secrete can damage the cell wall of the roots of *Platycodon grandifloras*, rendering them more susceptible to diseases (Lepas et al., 2025). The increase in cell cycle-*Caulobacter* genes is associated with the decomposition of soil organic matter, and such functional abnormalities may reduce the efficiency of mineralizing organic P (Zhao et al., 2025). This would be a logical consequence of the decrease in the contents of TP and AP.

Total phosphorus (TP) and available phosphorus (AP) showed a significantly positive correlation with the relative abundance of nucleotide sugar biosynthesis-related genes, but a significantly negative correlation with the abundance of *Caulobacter* cell cycle-related genes, indicating phosphorus availability affects key microbial metabolisms. Nucleotide sugar synthesis consumes substantial phosphorus; when TP and AP are sufficient, microorganisms acquire more phosphorus, which drives the upregulated expression of related genes and accelerates carbohydrate nucleotideylation. A high-phosphorus environment, however, disturbs the cell cycle of *Caulobacter* and disrupts its metabolic homeostasis. Additionally, enhanced nucleotide sugar synthesis may affect soil phosphorus retention, while the disrupted cell cycle of *Caulobacter* might reduce phosphorus transformation and exert a certain impact on phosphorus uptake by plants (Materu et al., 2024; Saini et al., 2024).

Continuous cropping duration exerted a distinct selective effect on the composition of soil fungal trophic functional groups. The significant enrichment of pathotrophic fungi in soils under 6-year continuous cropping (B) could likely be attributed to the alterations in soil microenvironment induced by long-term continuous cropping. The proliferation of pathogenic fungi generally indicates an elevated risk to soil health, and their positive correlation with cropping duration may serve as a critical microbial driver of the exacerbation of plant diseases in continuous cropping obstacles. This speculation is consistent with the observed phenomenon of aggravated root rot that occurred under prolonged continuous cropping (Schön et al., 2023). The marked increase in saprotrophic fungi in soils under 4-year (A) and 6-year (B) continuous cropping suggests that long-term continuous cropping can provide more favorable growth conditions for saprotrophic fungi by promoting organic matter accumulation or modifying carbon source availability. As the core functional group responsible for soil organic matter decomposition, saprotrophic fungi may accelerate the turnover efficiency of elements such as carbon and nitrogen. However, potential trade-offs between saprotrophic processes and plant nutrient competition under long-term continuous cropping warrant further attention (He et al., 2023). Although the variation in symbiotrophic fungi was not statistically significant, these fungi enhance nutrient uptake through symbiotic associations with plant roots (Teng et al., 2021). Their relative stability under long-term continuous cropping may indicate self-regulation of the soil ecosystem, yet their functional effectiveness requires comprehensive evaluation in conjunction with examination of both mycorrhizal colonization rates and plant growth parameters.

### 4.4 Effects of the years of continuous cropping on community succession and its ecological significance

The PCoA showed that the fungal community structures under the first cropping (CK) and continuous cropping for one stubble period (A) treatments were similar, while there was significant differentiation in the continuous cropping for two stubble periods (B). This was consistent with the cumulative nature of continuous cropping effects. In the early stage (the A treatment), there were only slight changes in the soil physicochemical properties, and the allelopathic effect of root exudates had not yet reached the threshold at which it can have observable biological effects; therefore, the community remained relatively stable (Ferrenberg et al., 2014; Yang et al., 2023; Tao et al., 2022). This transformation is typically accompanied by a decrease in the turnover of the soil carbon pool and a decrease in disease resistance (Hannula et al., 2017). This succession concept clarifies the phased regulation of continuous cropping obstacles. Short-term continuous cropping can be alleviated by optimizing water and fertilizer management, while long-term continuous cropping requires simultaneous improvement of the soil physicochemical properties and the microbial community structure.

### 4.5 Co-occurrence network analysis

The co-occurrence network results revealed the significant impact of the treatments on the interaction patterns of the fungal community. Treatment group A had the densest associations with fungal genera, suggesting that the environmental conditions under this treatment were overall more conducive to the growth of various fungi and their beneficial interactions, potentially promoting the species richness and functional diversity of the community. *Atractiella* is a genus of plant root fungal endophytes with a wide range of hosts. Previous research has shown that it can significantly promote plant growth and photosynthetic efficiency, and plays an important ecological role in plant nutrient absorption and growth promotion, as it is one of the key taxa involved in plant–microbe interactions in the soil microbial community (Duan et al., 2024). *Pisolithus* is a genus of ectomycorrhizal fungus that can form symbiotic relationships with various trees, including pines and eucalyptus. It enhances plant growth vitality by expanding the root absorption area and promoting the absorption of nutrients such as phosphorus, and it commonly occurs in barren, acidic, or heavy metal-contaminated soils. Its association with treatment groups A and B reflects the key role of the symbiotic interactions between mycorrhizal fungi and plants in maintaining soil ecological functions under natural conditions (Oliveira et al., 2024). *Bacidia*, as a lichen-forming fungal genus, is a pioneer in many ecosystems. It can grow on barren substrates, such as rocks and tree bark, decompose minerals by secreting organic acids to promote soil formation, and participate in carbon and nitrogen cycles. Its association with treatment groups CK and A reflects the key role of lichen fungi in soil initial development and ecological functions under natural conditions (Allen et al., 2021).

## 5 Conclusion

The present study showed that continuous cropping caused soil acidification and a decrease in the contents of total phosphorus, available phosphorus, and available potassium. Thus, this promoted a targeted nutrient deficiency–acidification intensification cycle, which directly drove the imbalance of the fungal community. This change resulted in an increase in acid-tolerant pathogens and a decrease in beneficial fungi. In turn, the potential functions of the fungal community exhibited shifts toward increased disease incidence, enrichment of pathotrophic fungi and saprotrophic fungi, and abnormal carbon metabolism. Finally, these changed significantly inhibited the growth of *Platycodon grandiflorus* and reduced its root length, root diameter, yield, and content of alcohol-soluble extract. These changes also increased the incidence rate and disease index of root rot. Among the observed fungal community changes, the enrichment of *Fusarium* was a key factor that stimulated root rot. Adjusting the soil pH, increasing the application of P and K fertilizers, and inoculating functional fungal agents have the potential to effectively alleviate the problems associated with continuous cropping of *Platycodon grandiflorus*.

Future research should explore the specific mechanisms of different regulatory measures on the rhizosphere microecosystem of this crop, such as the optimal combination of different pH adjustment methods, the rates of applying P and K fertilizer, and the types of functional fungal agents. In combination with molecular biology techniques, the molecular mechanism of the interaction between the fungal community and the growth of *Platycodon grandiflorus* under continuous cropping can be examined, enabling the validation of the potential functional shifts through metatranscriptomic or metaproteomic approaches. This would provide a stronger empirical basis and practical support for building a more efficient and precise prevention and control system to minimize the obstacles to continuous cropping of *Platycodon grandiflorus*. Such research would help the *Platycodon grandiflorus* industry to achieve sustainable and high-quality development.

## Data Availability

The metagenomic data in this study have been submitted to the NCBI database and specifically deposited in the NCBI BioProject platform, with the valid accession number PRJNA1335859 and the public retrieval link: https://www.ncbi.nlm.nih.gov/bioproject/PRJNA1335859. The sequencing data generated in this study have been deposited in the NCBI Sequence Read Archive (SRA) under the valid accession number SRP629296, and the accession numbers corresponding to each sample sequence are SRR35653706 - SRR35653717.

## Footnotes

1
https://github.com/OpenGene/fastp

2
https://github.com/voutcn/megahit

3
https://github.com/hyattpd/Prodigal

4
http://weizhongli-lab.org/cd-hit/

5
https://github.com/ShujiaHuang/SOAPaligner

6
https://github.com/bbuchfink/diamond

7
https://www.majorbio.com/tools
