# Supplementary figures and images for: Soil acidification and nutrient imbalance mediate fungal community degradation, a key driver of continuous cropping obstacles in Platycodon grandiflorus
Source: Front Microbiol. 2025 Nov 26;16:1716243. doi: 10.3389/fmicb.2025.1716243 (PMC12689964; doi:10.3389/fmicb.2025.1716243)

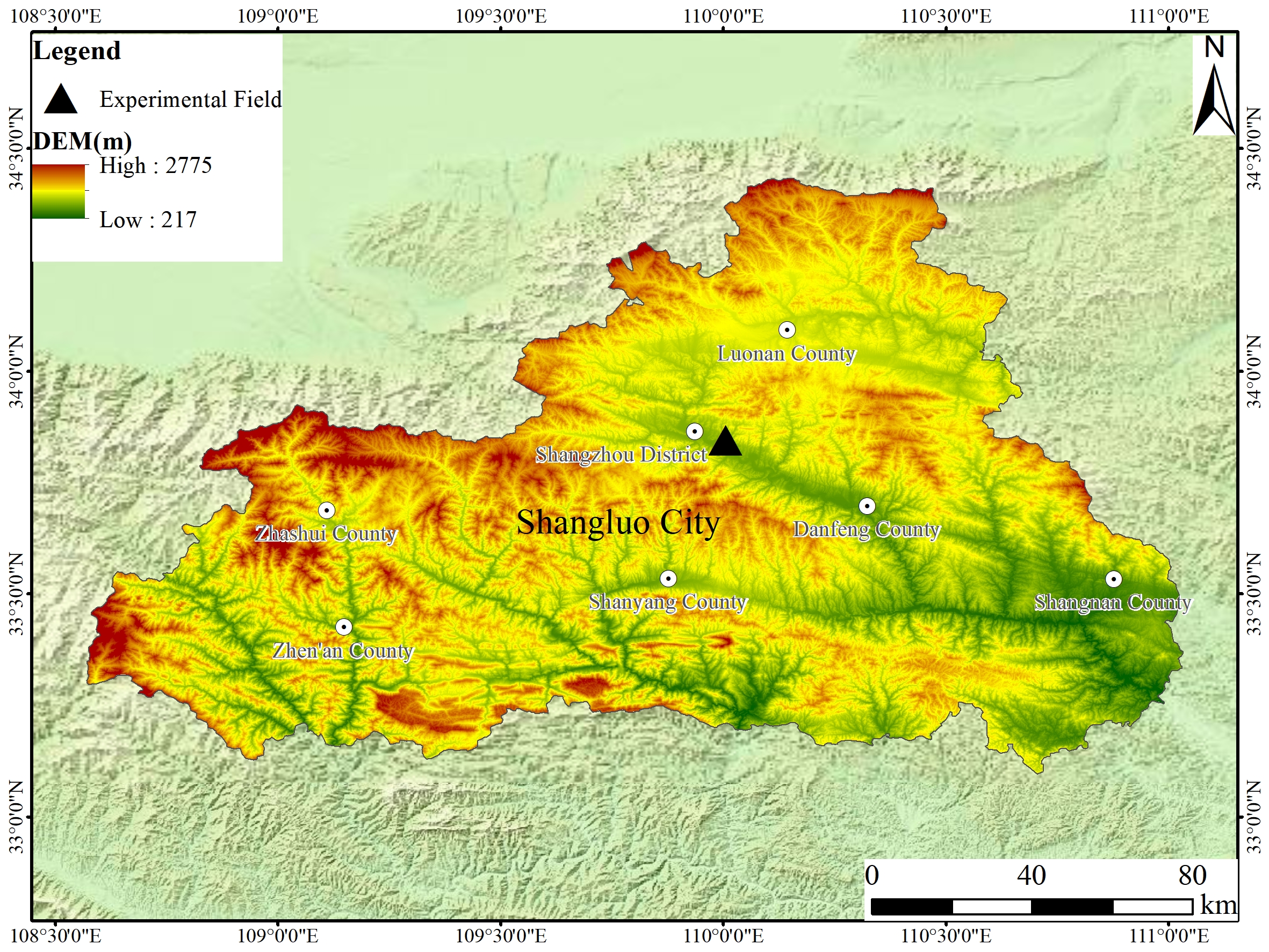

Supplement: Supplementary file 1 [file Image_1.jpeg]

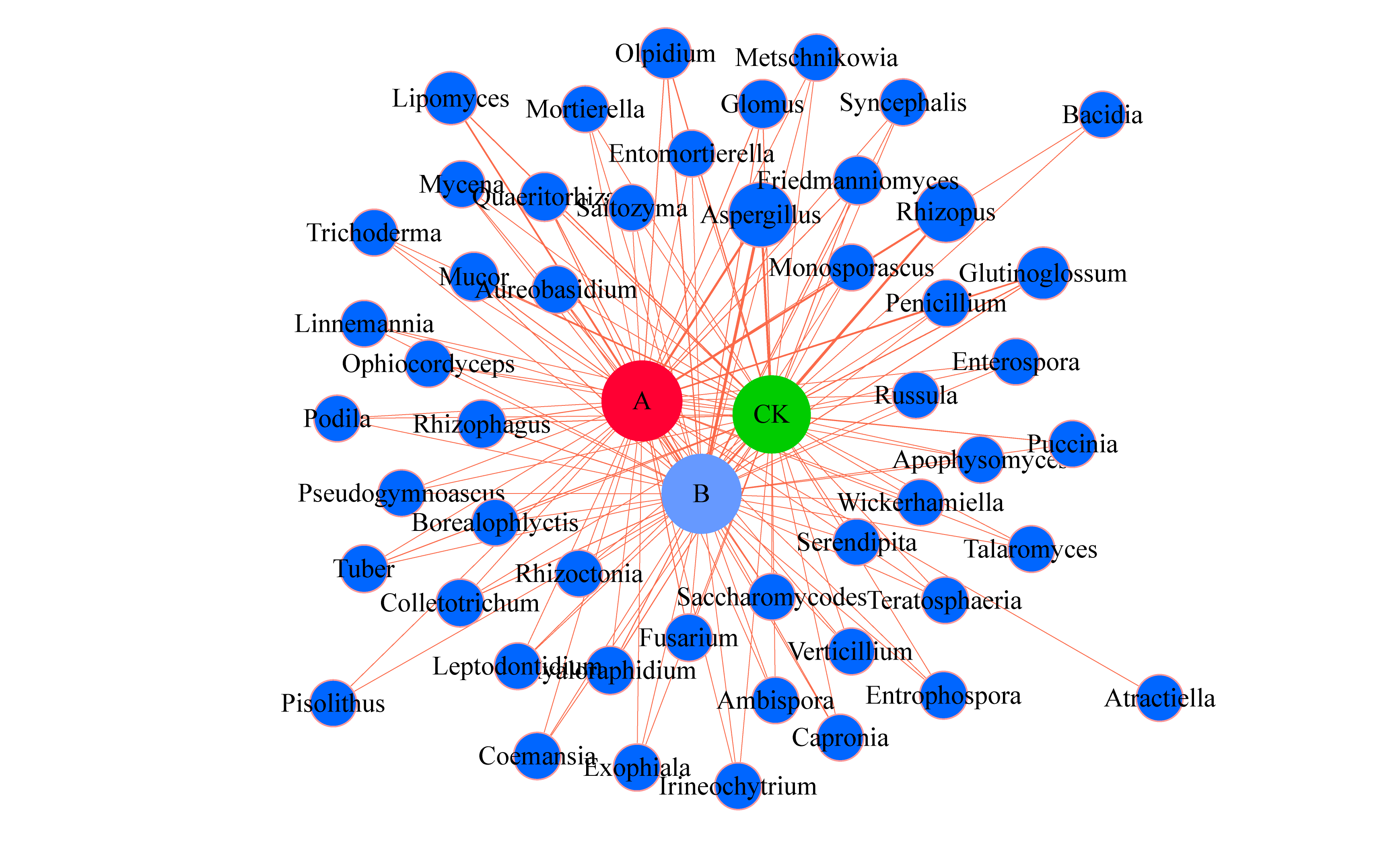

Supplement: Supplementary file 2 [file Image_2.tif]
