# Supplementary material for: Soil acidification and nutrient imbalance mediate fungal community degradation, a key driver of continuous cropping obstacles in Platycodon grandiflorus
Source: Front Microbiol. 2025 Nov 26;16:1716243. doi: 10.3389/fmicb.2025.1716243 (PMC12689964; doi:10.3389/fmicb.2025.1716243)
Supplement: Supplementary file 3 [file Table_1.docx]

Table S1. Experimental treatments of *Platycodon grandiflorus* based on continuous cropping years

| Treatment | Continuous cropping years of *Platycodon grandiflorus* | Planting pattern (2019–2024) |
| --- | --- | --- |
| CK | 2 years | Corn (2019–2022) → *Platycodon grandiflorus* (2023–2024) |
| A | 4 years | Corn (2019–2020) → *Platycodon grandiflorus* (2021–2024) |
| B | 6 years | *Platycodon grandiflorus* (2019–2024) |
