# Supplementary material for: Soil acidification and nutrient imbalance mediate fungal community degradation, a key driver of continuous cropping obstacles in Platycodon grandiflorus
Source: Front Microbiol. 2025 Nov 26;16:1716243. doi: 10.3389/fmicb.2025.1716243 (PMC12689964; doi:10.3389/fmicb.2025.1716243)
Supplement: Supplementary file 4 [file Table_2.docx]

Table S2 FUNGuild functional prediction in the rhizosphere soil of *Platycodon grandiflorus* under different continuous cropping years

| Trophic mode | Pathotroph (%) | Saprotroph (%) | Symbiotroph (%) |
| --- | --- | --- | --- |
| CK | 154.77±14.34b | 735.58±70.89b | 193.94±10.36a |
| A | 134.76±17.29b | 883.34±22.86a | 181.88±15.89a |
| B | 186.53±17.46a | 813.29±43.42a | 205.89±20.30a |

The data are the means±standard error (n=4). Different letters means significantly different based on *P*<0.05. CK, 2-year continuous cropping of *Platycodon grandiflorus*; A, 4-year continuous cropping of *Platycodon grandiflorus*; B, 6-year continuous cropping of *Platycodon grandiflorus*.
